# Prevalence of post-traumatic stress disorders and associated factors one month after the outbreak of the COVID-19 among the public in southwestern China: a cross-sectional study

**DOI:** 10.1186/s12888-021-03527-1

**Published:** 2021-11-04

**Authors:** Lei Lei, Hongyi Zhu, Yi Li, Tao Dai, Shouju Zhao, Xiaochao Zhang, Xiaoluzi Muchu, Shaoyu Su

**Affiliations:** 1 Department of Pediatric Intensive Care unit Nursing, West China Second University Hospital, Sichuan University/West China School of Nursing,Sichuan University, No. 20, Section 3, Renmin South Road, Chengdu, Sichuan China; 2 Key Laboratory of Birth Defects and Related Diseases of Women and Children (Sichuan University), Ministry of Education, Chengdu, Sichuan China

**Keywords:** COVID-19, PTSD, Exposure level

## Abstract

**Background:**

The Coronavirus Disease 2019 (COVID-19) pandemic has rapidly spread across the whole world and brought strong psychological impact. This study aimed to evaluate the prevalence of post-traumatic stress disorders (PTSD) in the general people of southwestern China and associated factors 1 month after the outbreak of the COVID-19.

**Methods:**

This study was started on 4–10 Feb 2020 based on online survey. The present work was carried out in the provinces of southeastern China, including Sichuan Province, Guizhou Province, Yunnan Province, and Chongqing City.1593 respondents aged 18 years and above administered to this study. Data on whether they have experienced confirmed or suspected COVID-19 of themselves/family members/acquaintances were also collected, and based on ‘yes’ answers, the number of affected individuals (via COVID-19) were categorized into four exposure levels i.e.*,* non-affected, less, moderately, or significantly affected*.* The civilian version of the PTSD checklist and the self-reported information about COVID-19 were used.

**Results:**

The prevalence of PTSD was approximately 25.2%(*n* = 401/1593). The chances of developing PTSD were 6.053(OR = 6.053, 95% CI 1.394 to 26.280) or 3.673(OR = 3.673, 95% CI 1.738 to 7.765) times higher among respondents who had been significantly and moderately affected than those who had not been affected, accordingly. Male (OR = 1.484, 95% CI 1.147 to 1.920),younger age individuals (40 ~ 49 age group/<30 age group, OR = 0.395, 95% CI 0.258 to 0.606) and health care workers (OR = 1.788, 95% CI 1.155 to 2.277) were at higher risk of developing PTSD.

**Conclusion:**

Our findings highlight that a positive correlation between the pandemic and PTSD. It is urgent to establish a screening and prevention systems for the population who are significantly exposed to COVID-19,and provide different psychological intervention strategies for different groups.

## Background

COVID-19 outbreak was caused by a novel and highly pathogenic strain of coronavirus. The disease was first reported in December 2019 in Wuhan city of China and later on Beijing announced that the virus could transfer from human-to-human on Jan 20, 2020. In this view, the Chinese government had implemented a lockdown (on Thu Jan 23rd, 2020) in Wuhan and other linked cities around Wuhan. The provinces of southwestern China i.e.*,* Guizhou, Sichuan, Yunnan, and Chongqing city, had started first-level reactions for the main public health emergencies since Jan 24, 2020.On Mar 2nd, 2020, a total number of 2915 COVID-19 associated deaths and 88,948 COVID-19 diagnosed cases in China and 128 deaths and 8774 cases of COVID-19 were reported by WHO in 64 other countries.

COVID-19 has totally affected people’s life. The most direct impact was social isolation and the panic of being infected, and a series of indirect effects followed by. The citizens were encouraged by policy to self-quarantine at home in order to minimize the risk of viral spreading. So, the people who should have been reunited with their family during the Spring Festival, which is the most important one, was failed. Some people even didn’t have the last chance to meet their loved ones. Many industries such as catering, entertainment and tourism were facing the challenge of survival. The confirmed or suspected COVID-19 patients were in danger of losing life. Healthcare professionals rushed to fight with the virus, and countless community workers and volunteers were also in the front line of the epidemic. Information about COVID-19 pandemic have been updated daily for the public through a variety of medium. At the beginning of the COVID-19 outbreak, the lack of specific treatments, limited knowledge of the virus and increasing death rates had further tensing the nervous of the society.

Post-traumatic stress disorder (PTSD) is a psychiatric syndrome that is developed by exposing to an extremely threatening or horrific event or series of events according to International Classification of Diseases,11th Revision (ICD-11) [1].COVID-19 pandemic unlike the normal natural disaster such as earthquake, hurricane and flood had the enormous power to threaten people’s lives immediately, but a substantial, potential and prolonged stressors with uncertain threat of health and life, unprecedented restrictions on movement and devastating economic consequences. The previous studies also mentioned that similar immense epidemics promoted development of PTSD [2–6]. A study of severe acute respiratory syndrome (SARS) showed that being infected or the threat of being infected can be a potentially traumatic event and elevates risk of developing PTSD [2, 5, 6]. After a year of the outbreak of the Ebola virus in Sierra Leone, among 27% of the participant met levels of clinical apprehension for PTSD and 16% met levels of possible PTSD [3]. In the Republic of Korea, 42.9% of survivors reported PTSD after 12 months of Middle East Respiratory Syndrome (MERS) [4].Kalin [7] enumerated several reasons to explain why the COVID-19 pandemic represents the perfect storm of stressors and traumatic events:1) a sense of uncontrollability and a sense of uncertainty about the future,2) concerns about contracting COVID-19, becoming gravely ill, and dying,3) worries about losing loved ones and friends, and the grief associated with real losses,4) prolonged physical separation and social isolation from family and friends,5) disruption of regular routines, including work and school for children,6) losses of jobs, business failures, and the profound economic consequences,7) lack of trust in leadership to effectively deal with the crisis. Thus, through this lens, the COVID-19 pandemic may be viewed as a valid traumatic event according to ICD-11.

PTSD is considered by some psychologists as a secondary consequence of the SARS-Cov-2 pandemic no matter for patients, healthcare professionals or general population [8]. Different populations may be disproportionately affected by COVID-19 pandemic both in terms of the level of exposure, as well as the amount and type of stressors they experience [9]. In a sample of Chinese healthcare professionals,40.2% indicated positive screens for significant PTSD symptoms from Feb 23 to March 5, 2020 [10]. The prevalence of clinically-relevant PTSD was 30.8% in a 2500 invited Chinese university students in a survey between Feb 12 and 17, 2020 [11],and 25.2% of confirmed COVID-19 patients were reported symptoms of posttraumatic stress in a Jianghan Fangcang shelter of Wuhan city from Feb 15 to 22,2020 [12]. A study reported that 44.5% of people who were geographically located in Wuhan were associated with severe symptoms of PTSD among 9225 participants after 1 month of COVID-19 outbreak in Hubei province [13]. At present, most of the study mentioned above focused on exploring the psychological state of healthcare professionals, patients and people near by the hotspots, the mental health condition of every ordinary person under the epidemic is still worthy of attention. In addition, at present, older age, male, having been isolated, knowing people who had been isolated, people who had recent epidemic area contact history, those at high risk of infection or with poor sleep quality were reported associated with higher prevalence of PTSD during the COVID-19 outbreak [11, 14–16].PTSD has brought about substantial medical and economic burden [17],and high suicide rates [18]. Under the epidemic situation of COVID-19, PTSD was not only reported to exacerbate and deteriorate of pre-existent mental symptoms [19],but people with mental diseases were more likely to develop PTSD [20]. Therefore, understanding and intervention of PTSD is highly essential as soon as possible.

Currently, control of the epidemic is still the dominant task across the world, and it seems that humanity will coexist with COVID-19 for a long time in the future. In order to a full understanding of the psychosocial responses induced by such infectious diseases from different aspects, to conduct an investigation in southwestern China, which hosts a population of over 160 million, is important. Hence, this study aimed to evaluate the prevalence of post-traumatic stress disorders (PTSD) in the general people of southwestern China and associated factors 1 month after the outbreak of the COVID-19.

## Methods

### Study settings

A prevalence study was started on 4–10 Feb 2020. The present work was carried out in the provinces of southeastern China, including Sichuan Province, Guizhou Province, Yunnan Province, and Chongqing City.

### Study participants and sampling

In the existing study, a snow ball sampling was employed for the participant’s selection, using an online questionnaire that can be accessed through https://jinshuju.net/f/Szvar5. The data collection was carried out by six researchers by sharing the access link to the WeChat (chatting app, most frequently used app in China) groups, comprising different people. As a result, we got 1987 responses, while a total of 1593 participants was incorporated for the last statistical evaluation after the omission of the responses that were found to be not meeting the recruitment criteria (age and location-based). Some responses data were found to be incomplete and some were with understandably false answers, while the ratio of the effective response was 80.2%. The quality control methods were employed in the current study which comprised the conditions that a similar IP address could be used only one time with suitable time duration to give each answer. Answering the questioner in less than 2 min the responses were considered invalid. The sample involved only adults aged no less than 18 years and above. All procedures involving human participants in this study were in accordance with the ethical standards of the the Medical Ethics Committee of West China Second Hospital of Sichuan University (2020011), and the 1964 Helsinki Declaration and its later amendments. Participation was anonymous and provided informed consent.

### Assessment and measurement

#### Measurement of PTSD

The measurement of PTSD was performed via 17 items of self-reported PCL. The PCL is a standardized scoring scale for PTSD, containing 17 items that resemble the significant detection and Statistical Manual of Mental Disorders-IV PTSD indications. A total indication-based severity score (range = 17 to 85) was achieved by adding the scores of the 17 items. It had Likert response options ranged from ‘not at all’ to ‘extremely’ and a cut-off point greater or equal to 50 i.e.*,* garbage landslide victims had PTSD signs [21]. Herein, we modified this instrument with the help of a reported study on Somali and Oromo Ethiopians in Minnesota [22]. It was found to be with elevated inner stability, reliability, and a closed association with PTSD detection. The reliability evaluation was performed for the PCL questionnaire (Amharic version) which was found to be with an elevated score i.e.*,* Cronbach’s α = 0.94.

#### Sociodemographic characteristics

The sociodemographic variables such as gender (male or female); age (less than 30, 30 ~ 39, 40 ~ 49, and greater or equal to 50 years); the level of qualification was categorized as ‘junior, middle school and below’, ‘high school’, and ‘university-level’; marital status, classified as ‘married’, ‘divorced’, and ‘single’; and occupational status, categorized as ‘Student’, ‘Agriculture, forestry, fishery and animal husbandry’, ‘Healthcare professionals’, ‘Police, firefighters, soldiers’, ‘Accommodation, catering, entertainment, tourism’, ‘Manufacturing, construction industry, transportation industry’ and ‘not working’, ‘Financial industry’, ‘Technical service industry’, and ‘Others’. Furthermore, the residential place (rural or urban) was also involved. Average income per mouth (in Chinese yuan [CNY], one CNY is equivalent to 0.15 US dollars) was categorized as ‘less than 1500’, ‘1500 ~ 2999’, ‘3000–5999’, ‘6000–8999’, and ‘greater or equal to 9000’.

#### Self-assessment knowledge regarding COVID-19

To examine the self-assessment level of knowledge regarding COVID-19, contributors were questioned, ‘about the level of knowledge about COVID-19 (self- perceived)? ’, and the contributors selected a score from 1 to 5, as shown in the questioner.

#### Self-perceived health condition

The participants were asked questions regarding their health condition (self- perceived) and the participants select a score from 1 to 5 that ranged from “very bad” health condition to “very good” health condition.

#### Worries regarding been infected

To evaluate the participants that are worried or not regarding being affected,’ and the participants selected a score from 1 to 3 score that ranged from “not worried at all” to “very worried”.

#### Property damage affected by COVID-19

To evaluate the property damage affected by COVID-19, questions were asked from participants, and the participants selected ‘0’, ‘less than 5000’, ‘around 5000 to 9999’, ‘around 10,000 to 29,999’ and ‘greater than 30,000’ Chinese Yuan [CNY].

Social support provided by community or government agencies.

The questions were asked from participants for the evaluation of the social (financial or psychological) support provided by government agencies, and the yes or no was the response options.

#### Information affected by COVID-19

The five questions were asked about their information affected by COVID-19 i.e.*,* Have you experienced confirmed infection or suspected infection? Have your family experienced confirmed infection or suspected infection? Have you or your family experienced designated isolation? Have you or your family experienced home isolation? Have your acquaintances experienced confirmed infection or suspected infection? The yes or no was the response options.

### Statistical analysis

The collected data were statistically analyzed via statistical software SPSS-V.19.0. categorical variables were represented as a number (percentile). In the current study, categorical variables were evaluated by the χ^2^ test. The *p-value* > 0.05 was found to be statistically considerable. A binary logistic regression analysis was used for the evaluation of the correlation between independent and dependent variables. PTSD linked factors were designated during the univariate analysis with a *p-value* < 0.05 for further analysis in the binary logistic regression analysis. In the binary logistic regression analysis, variables with *p-value* < 0.05 at 95% CI with OR were regarded as statistically important.

## Result

### Prevalence of PTSD

Out of the total 1593 participants, the prevalence of PTSD was 25.2%(*n* = 401/1593).

### Participant sociodemographic characteristics

The features of the participants included in this study are presented in Table 1. Most of the interviewees were female i.e., 976 with a percentage of 61.3%. The average age of the interviewees was 32.3 (SD ±9.8) years, the age of 77.1% (1228) of respondents were in the range of approximately 18 to 39 years. 80.7% (1285) of candidates were educated and from university; 56.4% (898) were married. Considering the status of employment, 16.1% of respondents were working in the manufacturing and construction industries as well as transportation. Almost 85.5% of these respondents were living in urban areas and around 60.6% (966) were belong to the Sichuan Province. The average income of 58.6% (933) was lower than 6000 Yuan.

**Table 1:** Comparison of prevalence of PTSD in different socio-demographic characteristics

Variables	All participants (*N* = 1593)	Positive PTSD (*n* = 401)	Negative PTSD (*n* = 1192)	df	χ^2^	*p* value
	*N*(%)	*n*(%)	*n*(%)			
**Sex**						
Female	976 (61.3)	222 (22.7)	754 (77.3)	1	7.878	0.005
Male	617 (38.7)	179 (29.0)	438 (71.0)			
**Age group (years)**						
< 30	749 (47.0)	221 (29.5)	528 (70.5)	3	23.076	<0.001
30 ~ 39	479 (30.1)	119 (24.8)	360 (75.2)			
40 ~ 49	257 (16.1)	38 (14.8)	219 (85.2)			
≥ 50	108 (6.8)	23 (21.3)	85 (78.7)			
**Education level**						
Junior, middle school and below	109 (6.8)	24 (22.0)	85 (78.0)	2	9.062	0.011
High school	199 (12.5)	34 (17.1)	165 (82.9)			
University-level	1285 (80.7)	343 (26.7)	942 (73.3)			
**Marital status**						
Single	651 (40.9)	182 (28.0)	469 (72.0)	2	4.928	0.085
Married/cohabiting	898 (56.4)	207 (23.1)	691 (76.9)			
Divorced/widowed	44 (2.8)	12 (27.3)	32 (72.7)			
**Occupational status**						
Student	226 (14.2)	62 (27.4)	164 (72.6)	8	16.199	0.040
Agriculture, forestry, fishery and animal husbandry	38 (2.4)	8 (21.1)	30 (78.9)			
Health care workers	251 (15.8)	83 (33.1)	168 (66.9)			
Police, firefighters, soldiers	35 (2.2)	12 (34.3)	23 (65.7)			
Accommodation, catering, entertainment, tourism	83 (5.2)	21 (25.3)	62 (74.7)			
Manufacturing, construction industry, transportation industry	257 (16.1)	53 (20.6)	204 (79.4)			
Financial industry	246 (15.4)	56 (22.8)	190 (77.2)			
Technical service industry	210 (13.2)	53 (25.2)	157 (74.8)			
Others	247 (15.5)	53 (21.5)	194 (78.5)			
**Region**						
Rural	231 (14.5)	54 (23.4)	177 (76.6)	1	0.463	0.566
Urban	1362 (85.5)	347 (25.5)	1015 (74.5)			
**Area**						
Sichuan Province	966 (60.6)	226 (23.4)	740 (76.6)	3	7.114	0.068
Chongqing City	211 (13.3)	50 (23.7)	161 (76.3)			
Guizhou Province	206 (12.9)	62 (30.1)	144 (69.9)			
Yunnan Province	210 (13.2)	63 (30.0)	147 (70.0)			
**Average income**						
< 1500	89 (5.6)	26 (29.2)	63 (70.8)	4	9.143	0.058
1500 ~ 2999	259 (16.3)	54 (20.8)	205 (79.2)			
3000 ~ 5999	585 (36.7)	152 (26.0)	433 (74.0)			
6000 ~ 8999	347 (21.8)	89 (25.6)	258 (74.4)			
≥ 9000	313 (19.6)	80 (25.6)	233 (74.4)			

According to univariate analysis, the occurrence of PTSD was considerably elevated in male i.e., 29.0% relative to female (22.7%; χ2 = 7.878, *p* = 0.005), as presented in Table 1. There was a considerable variation in the age groups regarding the occurrence of PTSD i.e., χ2 = 23.076, p<0.001. The PTSD occurrence was considerably elevated in the group (with age < 30) relative to group (having approximately 40 to 49 years age) i.e., χ2 = 21.687, p<0.001, the occurrence of PTSD was considerably elevated in around 30 to 39 years age group than 40 to 49 years age group (χ2 = 10.082, *p* = 0.001). There was a considerable variation in PTSD occurrence at educational levels i.e., χ2 = 9.062, *p* = 0.011, the PTSD prevalence was considerably elevated in those who obtained higher education in comparison with those who received education from secondary education i.e., χ2 = 7.620, *p* = 0.006. The PTSD prevalence was considerably different in the respondents having different occupational status i.e., χ2 = 16.199, *p* = 0.040, and the incidence of PTSD were significantly elevated in health-care workers than those who were working in construction industries and transportation i.e., χ2 = 10.032, *p* = 0.002 and financial industry i.e., χ2 = 6.547, *p* = 0.012.

### Participant information associated with COVID-19

The information of participants correlated with COVID-19 is presented in Table 2. In participants, 41.6% (663) participants had knowledge about COVID-19 (4 grade), 50.8% (810) said that they were good state of health (4 grade), and 52.9% (843) were little worried about their health as well as their family health considering COVID-19. Post COVID-19 outbreak, a property of around 15.3% was damaged beyond 10,000 Yuan. In all participants, 13.6% (217) revealed that financial support was given to them by the community or government. The government and communities also provide psychological counseling to 40.4% (643) participants.

**Table 2:** Comparison of prevalence of PTSD in different participant information associated with COVID-19

Variables	All participants (*N* = 1593)	Positive PTSD (*n* = 401)	Negative PTSD (*n* = 1192)	df	χ^2^	*p* value
	*N* (%)	*n* (%)	*n* (%)			
**Self-evaluated level of knowledge about COVID-19**						
Not at all	11 (0.7)	4 (36.4)	7 (63.6)	4	13.069	0.011
Low	39 (2.4)	8 (20.5)	31 (79.5)			
Medium	312 (19.6)	56 (17.9)	256 (82.1)			
Well	663 (41.6)	172 (25.9)	491 (74.1)			
Very well	568 (35.7)	161 (28.3)	407 (71.7)			
**Self-perceived health condition**						
Very bad	6 (0.4)	3 (50.0)	3 (50.0)	4	30.171	<0.001
Bad	35 (2.2)	8 (22.9)	27 (77.1)			
Not too bad	398 (25.0)	85 (21.4)	313 (78.6)			
Good	810 (50.8)	181 (22.4)	629 (77.6)			
Very good	344 (21.6)	124 (36.1)	220 (63.9)			
**Are you worried that you or your family may be infected?**						
Not at all	297 (18.6)	104 (35.1)	193 (64.9)	2	19.199	<0.001
A little worried	843 (52.9)	198 (23.5)	645 (76.5)			
Very worried	453 (28.4)	99 (21.9)	354 (78.1)			
**Property damage affected by COVID-19**						
0	500 (31.4)	151 (30.2)	349 (69.8)	4	16.496	0.002
< 5000	572 (35.9)	132 (23.1)	440 (76.9)			
5000 ~ 9999	277 (17.4)	50 (18.1)	227 (81.9)			
10,000 ~ 29,999	148 (9.3)	42 (28.4)	106 (71.6)			
> 30,000	96 (6.0)	26 (27.1)	70 (72.9)			
**Have you received any financial support or practical help from the community/government agencies?**						
Yes	217 (13.6)	54 (24.9)	163 (75.1)	1	0.011	0.916
No	1376 (86.4)	347 (25.2)	1029 (74.8)			
**Have you received any psychological support or consolation from the community/government agencies?**						
Yes	643 (40.4)	148 (23.0)	495 (77.0)	1	2.660	0.112
No	950 (59.6)	253 (26.6)	697 (73.4)			

According to univariate analyses, the prevalence of PTSD was significantly different in the self-evaluated levels of knowledge about COVID-19 (χ2 = 13.069, *p* = 0.011), as presented in Table 2, the PTSD was highly prevalent in those who believed that they had a significant knowledge regarding COVID-19 (grade 4 and 5) in comparison with a medium group (grade 3; χ2a = 7.567, pa = 0.006; χ2b = 11.716, pb = 0.001). The PTSD prevalence was considerably variant in the various self-perceived health conditions (χ2 = 30.171, *p*<0.001), and the prevalence of PTSD was considerably elevated in the group which had highly significant self-perceived health condition (5 grade), as compared to groups which had moderately self-perceived health conditions (3 grade; χ2 = 19.679, *p*<0.001) and significant self-perceived health condition, respectively (4 grade; χ2 = 23.310, *p*<0.001). The PTSD prevalence was considerably different in the level of worrying about being infected (χ2 = 19.199, *p*<0.001), and the prevalence of PTSD in those who were not worried was considerably elevated, as compared to those who had a little worried (χ2 = 19.199, *p*<0.001) and who were very worried (χ2 = 15.744, *p*<0.001). The prevalence of PTSD was considerably different in the different property damage level (χ2 = 16.496, *p* = 0.002), and the prevalence of PTSD was considerably elevated in those whose property had not been damaged, as compared to those whose property had been damaged beyond 5000 Yuan (χ2 = 6.967, *p* = 0.010) and around 5000 to 9999 Yuan (χ2 = 13.720, p<0.001), and the PTSD prevalence was significantly elevated in those whose property had been damaged up to 10,000 ~ 29,999 Yuan than those whose property had been damaged up to 5000 ~ 9999 Yuan (χ2 = 6.066, *p* = 0.019).

### Participant’s information affected by COVID-19

Table 3 represents the information of participants that have been affected by COVID-19. In all participants, 0.6% (10) have confirmed or suspected infection, and 0.4% (7) revealed that the confirmed or suspected infections were observed in their families. 1.8% (29) respondents and their family members were isolated while 18.0% (286) respondent or their family members have experienced home isolation.140(8.8%) experienced confirmed or suspected cases in their community, school, or workplace.

**Table 3:** Comparison of prevalence of PTSD in different participants affected by COVID-19

	All participants (*N* = 1593)	Positive PTSD (*n* = 401)	Negative PTSD (*n* = 1192)	χ^2^	*p* value
	*n*(%)	*n*(%)	*n*(%)		
Have you experienced a confirmed infection or suspected infection?					
Yes	10 (0.6)	8 (80.0)	2 (20.0)	13.264^a^	<0.001
No	1583 (99.4)	393 (24.8)	1190 (75.2)		
Have your family experienced confirmed infection or suspected infection?					
Yes	7 (0.4)	4 (57.1)	3 (42.9)	2.301^a^	0.129
No	1586 (99.6)	397 (25.0)	1189 (75.0)		
Have you or your family experienced designated isolation?					
Yes	29 (1.8)	13 (44.8)	16 (55.2)	6.058	0.028
No	1564 (98.2)	388 (24.8)	1176 (75.2)		
Have you or your family experienced home isolation?					
Yes	286 (18.0)	86 (30.1)	200 (69.9)	4.438	0.042
No	1307 (82.0)	315 (24.1)	992 (75.9)		
Have your acquaintances experienced confirmed infection or suspected infection?					
Yes	140 (8.8)	32 (22.9)	108 (77.1)	0.437	0.542
No	1453 (91.2)	369 (25.4)	1084 (74.6)		

^a^Correction for continuity χ^2^

According to univariate analysis, the PTSD prevalence was considerably elevated in participants having confirmed infection or suspected infection as compared to those participants who have not been experienced confirmed or suspected infection (χ2 = 13.264, *p*<0.001), as presented in Table 3. The PTSD was significantly prevalent in participant and their family members that were isolated in comparison with those that were not isolated (χ2 = 6.058, *p* = 0.028). PTSD was significantly prevalent in participant and their family members who have experienced home isolation than those who have not been experienced home isolation (χ2 = 4.438, *p* = 0.042).

Data on whether they have experienced confirmed or suspected COVID-19 or isolation of themselves/family members/acquaintances, and based on ‘yes’ answers, the number of affected individuals (via COVID-19) were categorized into four exposure levels i.e., non-affected, less, moderately, or significantly affected. Less affected represents that respondents have one ‘yes’ answer, and moderately affected represents that respondents have two‘yes’answers, and significantly affected represents that respondents have three or more than three‘yes’answers. The severity of PTSD was significantly different depending on the degree of experienced exposure of our respondents to COVID-19 (χ^2^ = 22.253, *p*<0.001), as presented in Table 4. The PTSD prevalence was considerably decreased in the non-affected group, as compared to the moderately (χ^2^ = 13.343, *p* = 0.001) and significantly affected groups (χ^2^ = 8.424, *p* = 0.010), and the PTSD prevalence was considerably reduced in a less affected group than moderately affected (χ^2^ = 13.547, *p* = 0.001) as well as significantly affected group (χ^2^ = 8.867, *p* = 0.008). The considerable variations were not observed between non-affected and less affected groups (χ^2^ = 0.207, *p* = 0.649), while the considerable variations were also not observed in moderately and significantly affected groups (χ^2^ = 0.523, *p* = 0.706).

**Table 4:** Comparison of prevalence of PTSD in different participant affected by COVID-19

	All participants (*N* = 1593)	Positive PTSD (*n* = 401)	Df	χ^2^	*p* value
	*n*(%)	*n*(%)			
Non-affected	1173 (73.6)	289 (24.6)	3	22.253	<0.001
Less affected	379 (23.8)	89 (23.5)			
moderately affected	32 (2.0)	17 (53.1)			
Significantly affected	9 (0.6)	6 (66.7)			

### Factors associated with PTSD among the participants

Binary logistic regression analysis was conducted to identify the correlation between independent variables and PTSD. In the previous univariate analysis, factors, such as sex, age, educational level, occupational status, self-evaluated information regarding COVID-19, self-perceived health condition, worried about being infected, damage of property, and the degree of being affected was included in the binary logistic regression model for controlling confounding effects.

The obtained results of the binary logistic analysis revealed that male sex, the group included less than 30 age participants, healthcare professionals, not worried about being infected, property not being damaged, moderately and significantly affected were considerably correlated with PTSD at a *p*-value< 0.05, as presented in Table 5. Male were more likely to develop PTSD (1.484 times), as compared with the female (OR = 1.484, 95% CI 1.147 to 1.920). The chances of PTSD were 0.395 in the 40 ~ 49 age group, as compared to < 30 age group (OR = 0.395, 95% CI 0.258 to 0.606). Respondents who were healthcare professionals were more likely to develop PTSD (1.788 times), as compared with those who were students (OR = 1.788, 95% CI 1.155 to 2.277). The probability of PTSD was 0.605 (OR = 0.605, 95% CI 0.442 to 0.827) and 0.591 (OR = 0.591, 95% CI 0.413 to 0.845) in respondents who had little and more worried about being infected, respectively, as compared to respondents who had not worried about being infected. The probability of PTSD was 0.531(OR = 0.531, 95% CI 0.362 to 0.778) in respondents whose property (5000 ~ 9999 yuan) had been damaged, as compared to the respondents whose property had not been damaged. The chances of developing PTSD were 3.673 times higher among respondents who had affected by moderately affected than those who had not been affected (OR = 3.673, 95% CI 1.738 to 7.765), and 6.053 times higher among respondents who had affected by very significantly affected than those who had not been affected (OR = 6.053, 95% CI 1.394 to 26.280).

**Table 5:** Factors associated with PTSD among the participants

Variables	Category	B	SD	WALD	p	OR	95% CI
Sex							
Female							
Male*		0.395	0.131	9.026	0.003	1.484	1.147 ~ 1.920
Age group (years)							
< 30							
30 ~ 39		−0.251	0.151	2.759	0.097	0.778	0.578 ~ 1.046
40 ~ 49*		−0.928	0.218	18.116	<0.001	0.395	0.258 ~ 0.606
≥ 50		−0.421	0.279	2.270	0.132	0.656	0.380 ~ 1.135
Education level							
Junior, middle school and below							
High school		−0.591	0.321	3.398	0.065	0.554	0.295 ~ 1.038
University-level		−0.132	0.278	0.225	0.635	0.877	0.508 ~ 1.511
Occupational status							
Student							
Agriculture, forestry, fishery and animal husbandry		0.044	0.473	0.009	0.926	1.045	0.413 ~ 2.641
Healthcare workers*		0.581	0.223	6.780	0.009	1.788	1.155 ~ 2.277
Police, firefighters, soldiers		0.494	0.424	1.359	0.244	1.639	0.714 ~ 3.762
Accommodation, catering, entertainment, tourism		0.035	0.338	0.011	0.917	1.036	0.535 ~ 2.008
Manufacturing, construction industry, transportation industry		−0.083	0.242	0.117	0.732	0.921	0.573 ~ 1.479
Financial industry		−0.022	0.237	0.008	0.927	0.979	0.614 ~ 1.559
Technical service industry		0.212	0.240	0.780	0.377	1.237	0.772 ~ 1.981
Others		0.238	0.250	0.909	0.340	1.269	0.778 ~ 2.070
Self-evaluated level of knowledge about COVID-19							
Not at all							
Low		−0.326	0.823	0.157	0.692	0.722	0.144 ~ 3.620
Medium		−0.551	0.727	0.575	0.448	0.576	0.139 ~ 2.395
Well		−0.153	0.716	0.046	0.831	0.858	0.211 ~ 3.491
Very well		−0.181	0.716	0.064	0.801	0.835	0.205 ~ 3.399
Self-perceived health condition							
Very bad							
Bad		−1.382	0.983	1.975	0.160	0.251	0.037 ~ 1.725
Not too bad		−1.194	0.893	1.789	0.181	0.303	0.053 ~ 1.744
Good		−1.192	0.889	1.798	0.180	0.304	0.053 ~ 1.734
Very good		−0.598	0.892	0.449	0.503	0.550	0.096 ~ 3.161
Are you worried that you or your family may be infected?							
Not at all							
A little worried*		−0.503	0.159	9.940	0.002	0.605	0.442 ~ 0.827
Very worried*		−0.526	0.182	8.319	0.004	0.591	0.413 ~ 0.845
Property damage affected by COVID-19							
0							
< 5000		−0.264	0.148	3.196	0.074	0.768	0.575 ~ 1.026
5000 ~ 9999*		−0.633	0.195	10.567	0.001	0.531	0.362 ~ 0.778
10,000 ~ 29,999		0.060	0.221	0.075	0.784	1.062	0.689 ~ 1.637
> 30,000		−0.022	0.266	0.007	0.934	0.978	0.581 ~ 1.647
Different degree of being affected							
Non-affected							
Affected by at least one aspect		0.037	0.145	0.066	0.797	1.038	0.781 ~ 1.380
Affected by at least two aspects*		1.301	0.382	11.606	0.001	3.673	1.738 ~ 7.765
Affected by three aspects and above*		1.801	0.749	5.777	0.016	6.053	1.394 ~ 26.280
Constant		0.810	1.016	0.635	0.426	2.247	

*P-value< 0.05, model fitness = 0.436 (Hosmer and Lemshow), <0.001 (Omnibus test), no multicolinearity (tolerance > 0.1and variance inflation factor (VIF) < 2)

*OR* odd ratio; *PTSD* post-traumatic stress disorder

## Discussion

To the best of our knowledge, this is the first study to investigate the prevalence and associated factors of PTSD among general population in southwestern China in about 1 month after the COVID-19 outbreak in China. The prevalence of PTSD was 25.2% from the result of this study. The current study with showed that males, adults less than 30 age, and health care workers were in the risk of developing higher PTSD. High level of exposure to COVID-19 were also found to be significantly associated with higher PTSD. Unexpected, there was a significant correlation between higher PTSD and that not worried about being infected and no damage to the property.

The prevalence of PTSD was 25.2% in our study. Before COVID-19 pandemic, a survey showed the weighted life-time prevalence of any mental disorders (excluding dementia) was 16.6% in China [23],and another research reported that 11.8% of adult survivors from 2008 Wenchuan Earthquake in southwestern China had probable PTSD after 8 years of the earthquake [24]. Comparing the PTSD prevalence rate before and after the pandemic, the obvious increase of this rate after the pandemic indicates a positive correlation between the pandemic and PTSD, and it is reasonable to assume that COVID-19 pandemic can be highly traumatic experiences. A recently study based on resting-state functional connectivity MRI (rs-fcMRI) also showed that COVID-19 pandemic was a crucial stressor to bring risks developing PTSD symptom [25]. The underlined result was consistent with the results of several recently published systematic review and meta-analyses. Surapon [26] searched articles including information from 32 different countries and 398,771 participants (general population) published from Jan 1, 2020, to Jun 16, 2020, and this period was the initial stage of global epidemic. Their results showed that the global prevalence estimate was 24.1% for post-traumatic stress symptoms. Another similar articles reported the pooled prevalence of post-traumatic stress symptoms was 28.34% [14] and 21.94% [27]. The research mentioned above showed that under the epidemic situation, the general population were at a high level of PTSD across countries and regions, suggesting the attention to PTSD related to COVID-19 should be increased globally and identifying related risk factors in terms of reducing the mental health burden of COVID-19 is crucial.

Under the COVID pandemic, some reports revealed a higher percentage of PTSD compared to the present study. Despite the different methodological methods used, our findings show that the pooled prevalence of PTSD is higher. Such as in the Italian population the PTSD prevalence was reported at around 29% in two weeks instantly post COVID-19 outbreak (from March 18 to 31,2020) [28]. Another report of the Chinese general population revealed that PTSD was significantly prevalent in 67.09% of the participants (self-reported) at the end of March 2020 [29]. Some reports revealed a lower percentage of PTSD percentage than the results obtained from the present study, such as the rate of COVID-19-associated PTSD was 17.7% in the Irish general population (the underlined survey was completed on April 52,020.) [30]. A study reported that COVID-19-associated PTSD symptoms were prevalent in a sample of adults living in Wuhan and surrounding cities in China and the underlined study also revealed that 7% of participators met the diagnostic criteria for PTSD between January 30 and February 8, 2020 [31]. In a sample of the Spanish population, PTSD symptoms were observed in 15.8% of participants (from March 21 to 28, 2020) [32]. Moreover, various races and cultural backgrounds, various cut-off points, different measurement, and past traumatic experience of individuals leads to the variations in the PTSD prevalence. The PTSD prevalence was significantly associated with the duration of the COVID-19 outbreak at which the survey was performed. In fact, PTSD symptoms usually appear months after the traumatic experience [33]. For example, in SARS the mental health was significantly impaired after the acute outbreak than in the initial phases [34], and after the SARS outbreak in 2003, the PTSD prevalence in survivors of SARS was 9.79% in their initial stage of recovery [35] and 25.6% at 30-months after SARS assessment [5]. So, with the development of the pandemic, how the prevalence of PTSD will change will become the next question for us to explore.

In the present study, the rate of PTSD prevalence was found to be higher in males relative to the female which showed consistency with the report in the Irish general population [30]. On the contrary, many studies evaluated the traumatic effects of COVID-19 and revealed that the females were at higher risk to develop PTSD symptoms [31, 32, 36]. Earlier reports on the outbreak revealed that the rate of PTSD prevalence was13.0–20.4% and 6.2–8.2% for women and men, respectively [37, 38] which shows that the chances of females developing PTSD were two times higher in comparison with males [39]. But in the condition of isolation and quarantine, men usually cannot express their feelings that may result in highly negative emotions.

In the present study, the PTSD prevalence is 29.5% in younger people (< 30 age) and the underlined result has been consistent with the results of PTSD (31.8%) in young adults (18–30 years) of U. S during the COVID-19 pandemic [40]. The present study revealed that in the < 30 age group the PTSD was significantly higher, as compared to the 40 ~ 49 years age group. The obtained results showed consistency with the reported study which revealed that younger age groups were more likely to develop psychological distress [41],and the diagnostic requirement for COVID-19-associated PTSD showed a significant correlation with younger age group [30] because young people have less life experience than middle-aged people, and it is difficult to deal with personal problems, such as completing school on time or finding a suitable job. In addition, young people use more social networking software and are exposed to more information related to the pandemic which may elevate the psychological burden of young people to a certain extent. Huang and Ni results also showed consistency with the underlined reports [42, 43].

Post outbreak, health care workers are fighting on the front line of protest, while students are strictly protected and isolated and are not allowed to go side and our obtained results revealed that health care workers are at higher risk of developing PTSD than students. A study has reported that the prevalence of PTSD was 2.7% in a sample of Chinese university students (home quarantined), and in this study, around 89.7% of students were quarantined at home [44]. The outbreak of the pandemic has put tremendous pressure on the medical system around the world. The front-line health workers who directly or indirectly deal with COVID-19 patients bear more risks and workload. Furthermore, the medical staff needs more attention and professional guidelines because of the lack of PPE at the beginning of the outbreak, working for long hours, at risk of been infected, the unknown future of the pandemic, separation from the family, and the psychological burden of staff in the medical system. A systematic review did not evaluate statistically considerable variations in PTSD and occupational stress of health care workers, as compared with none-health care workers until May 02, 2020 [45], and in the present study, the obtained results revealed that no considerable variation has been found in PTSD prevalence between health care workers and non-healthcare workers excepted for students.

Earlier studies have been revealed that worry about infection has been positively correlated with PTSD [30, 41, 46, 47]. But our finding revealed that a large number of participants were developing PTSD that was not worried about being infected, as compared to those who are little and more worried about being infected which might be due to limited information regarding COVID-19 in the initial phase of the outbreak and the risks may not be recognized. In addition, when a major traumatic event or loss occurs, people’s first reaction is to escape and not to believe that their lives will change, they tend not willing to admit that they are worried about being infected by SARS-CoV-2.

For ordinary people, that themselves, their family members, and someone they used to know had been confirmed/suspected with COVID-19 or nobody around them has been confirmed/suspected with COVID-19 are at different levels of developing PTSD. Among the participants, the PTSD prevalence was significantly elevated (80.0%) in participants who had experienced confirmed or suspected COVID-19. Another reported study revealed that in China the symptoms of post-traumatic stress were significantly prevalent in clinically stable patients (COVID-19 affected) i.e.*,* 96.2% [48]. The PTSD was significantly higher among individuals who were significantly exposed to COVID-19. Our obtained results show consistency with other results, for example, Sun et al. reported the study in which survey data was collected from a general sample of adults in the Chinese mainland, and the obtained data revealed that 4.6% of the participants showed symptoms suggesting more likely to develop PTSD. Moreover, the chances of PTSD prevalence was considerably elevated (18.4%) in a subsample of participants that were greatly exposed to COVID-19 (suspected or confirmed COVID-19) and also those who had close contact with a person infected with COVID-19 [49]. Recently, another report revealed that the participants that are significantly exposed to COVID-19 have a considerable correlation with moderately or severe symptoms of PTSD such as those participants that are the friend or family member of a health-care professional or a person having confirmed COVID-19 or having family member or friends that visited Wuhan [50]. So, in the present study, it has been suggested that in the early phase of the outbreak, different levels of psychological support should be provided to the people based on the level of exposure to COVID-19.

### Limitations

There are a few limitations of this study. Firstly, the specific detection tool was not used for COVID-19-associated PTSD and all complications associated with mental health were evaluated via self-report measures. Secondly, we adopted snowball sampling strategy. The snowball sampling strategy is not based on random selection of samples and does not truly reflect the actual pattern of the general population. Moreover, respondents had to use a computer or smartphone to respond, indicating that they may be more educated and socioeconomically stable than the population as a whole. At last, in this study, we just evaluated the symptoms of PTSD that were correlated with the current pandemic, and did not evaluate the pre-trauma factors which might crucially contribute to PTSD, as well as existing mental and physical illnesses apart from COVID-19.

## Conclusions

The results of this study provide the first data about the PTSD under the initial stage of COVID-19 carried out in southwestern China. Our findings highlight that a positive correlation between the pandemic and PTSD, and the high level of exposure, males, adults less than 30 age, and health care workers were more likely to report elevated PTSD. This found helps in the identification of the target population in the early phase of the pandemic. It is urgent to build a screening and prevention systems for the population who are significantly exposed to COVID-19, and underscore the need to provide different psychological intervention strategies for different groups of people according to their characteristics. Simultaneously, the results obtained in the present study could be combined with future studies for the comparison of PTSD prevalence during different periods, and assist to establish standard intervention strategy for mental disorder in a potential pandemic outbreak in the future.

## Data Availability

The datasets generated and/or analyzed during the current study are not publicly available because they are anonymized, though they are available from the corresponding author on reasonable request.
